# Xenotropic murine leukemia virus-related virus is not associated with chronic fatigue syndrome in patients from different areas of the us in the 1990s

**DOI:** 10.1186/1743-422X-8-450

**Published:** 2011-09-24

**Authors:** Mir A Ali, Janet K Dale, Christine A Kozak, Raphaela Goldbach-Mansky, Frederick W Miller, Stephen E Straus, Jeffrey I Cohen

**Affiliations:** 1 Medical Virology Section, Laboratory of Infectious Diseases, National Institute of Allergy and Infectious Diseases, National Institutes of Health, Bethesda, Maryland, USA; 2 Viral Biology Section, Laboratory of Molecular Microbiology, National Institute of Allergy and Infectious Diseases, National Institutes of Health, Bethesda, Maryland, USA; 3 Translational Autoinflammatory Disease Section, National Institute of Arthritis and Musculoskeletal and Skin Diseases, National Institutes of Health, Bethesda, Maryland, USA; 4 Environmental Autoimmunity Group, National Institute of Environmental Health Sciences, National Institutes of Health, Bethesda, Maryland, USA; 5 Current Address: Clinical Research Program, Division of Allergy, Immunology and Transplantation, National Institute of Allergy and Infectious Diseases, National Institutes of Health, Bethesda, Maryland, USA

**Keywords:** chronic fatigue syndrome, xenotropic murine leukemia virus-related virus, murine leukemia virus

## Abstract

**Background:**

In 2009, xenotropic murine leukemia virus-related virus (XMRV) was reported in 67% of patients with chronic fatigue syndrome (CFS) compared to 4% of controls. Since then numerous reports failed to detect XMRV in other cohorts of CFS patients, and some studies suggested that XMRV sequences in human samples might be due to contamination of these samples with mouse DNA.

**Results:**

We determined the prevalence of XMRV in patients with CFS from similar areas in the United States as the original 2009 study, along with patients with chronic inflammatory disorders and healthy persons. Using quantitative PCR, we initially detected very low level signals for XMRV DNA in 15% of patients with CFS; however, the frequency of PCR positivity was no different between patients with CFS and controls. Repeated attempts to isolate PCR products from these reactions were unsuccessful. These findings were supported by our observations that PHA and IL-2 stimulation of peripheral blood mononuclear cells from patients with apparently low levels of XMRV, which induced virus replication in the 2009 report, resulted in the disappearance of the signal for XMRV DNA in the cells. Immunoprecipitation of XMRV-infected cell lysates using serum from patients from whom we initially detected low levels of XMRV DNA followed by immunoblotting with antibodies to XMRV gp70 protein failed to detect antibody in the patients, although one control had a weak level of reactivity. Diverse murine leukemia virus (MLV) sequences were obtained by nested PCR with a similar frequency in CFS patients and controls. Finally, we did not detect XMRV sequences in patients with several chronic inflammatory disorders including rheumatoid arthritis, Bechet's disease, and systemic lupus erythematosus.

**Conclusions:**

We found no definitive evidence for XMRV DNA sequences or antibody in our cohort of CFS patients, which like the original 2009 study, included patients from diverse regions of the United States. In addition, XMRV was not detected in a cohort of patients with chronic inflammatory disorders.

## Background

Chronic fatigue syndrome (CFS) is characterized by debilitating, unexplained, persistent or relapsing severe fatigue of new onset that is not relieved by rest or reduction of activities. In addition, criteria for CFS require that patients concurrently have four or more of the following symptoms for ≥6 months (a) impaired memory or concentration, (b) sore throat, (c) tender cervical or axillary lymph nodes, (d), muscle pain, (e) multi-joint pain without redness or swelling, (f) headache, (g) unrefreshing sleep, or (h) post-exertional malaise. While a large number of infectious agents have been postulated to cause CFS, further studies have not confirmed these findings. In 2009, Lombardi et al. [1] first reported the presence of xenotropic murine leukemia virus-related virus (XMRV) in the blood of 67% of patients with CFS compared with 3.7% of control subjects. In a recent study, Lo et al. [2] reported the presence of murine leukemia virus (MLV)-related virus gene sequences in 86.5% of CFS patients and 6.8% of controls. The sequences amplified by nested PCR from these patients were distinct from XMRV reported by Lombardi et al. [1]. Recently, a number of other studies have failed to confirm this observation [3-10]. Recent studies have suggested that amplification of XMRV DNA in human samples is due to contamination of these samples with mouse DNA [11-15].

In view of the controversies linking CFS to MLVs among different laboratories, we tested our well characterized cohort of chronic fatigue syndrome patients that fulfilled the CDC case definition [16] for both XMRV and MLV-related viruses. We failed to find definitive evidence for XMRV DNA sequences or antibody in our cohort of CFS patients, which were from diverse areas of the United States, similar to the cohort reported in original 2009 study [1,17]. We did, however, detect a diverse set of MLV-related virus gene sequences at a similar frequency in CFS patients as in healthy individuals.

## Results

### A very weak signal is detected for XMRV in PBMCs from some patients with CFS, but the frequency of PCR positivity is not significantly different from controls

In the first set of experiments, we determined the frequency and level of XMRV DNA in blood obtained from cohort 1 which included patients with CFS (21-61 years), idiopathic chronic fatigue, other viral diseases, and healthy blood bank donor controls obtained from 1993-2007 (Table 1). As reported previously for patients with CFS [7-9], most of the patients and controls in the cohort were Caucasian women ages 40-45. Most patients and controls were from the Midwest or Southern United States; other patients were from the Northeastern and Western United States.

**Table 1:** Characteristics of Subjects in Cohort 1 Evaluated for XMRV.

	Chronic fatigue syndrome	Idiopathic chronic fatigue	Health blood donors	Non-CFS virus disease^1^
No. persons	61	6	9	3

XMRV+/-	9/52	0/6	0/9	0/3

Age, yrs	XMRV+/-			

Mean	44/43	41	35	41

Median	45/43	42	31	41

Range	30-54/21-61	28-61	16-54	20-56

White (%)	100/94	100	100	67

Women (%)	100/75	83	88	67

Mean yrs. of illness	5/6	5	NA	NA

Residential geographic zone^2 ^(%)				

Midwest	56/36	67	50	33

Northeast	0/13	0	0	0

South	22/36	33	50	67

West	22/15	0	0	0

CFS. chronic fatigue syndrome; NA, not applicable

^1^Non-CFS viral diseases: one case each of chronic active hepatitis B virus infection, echovirus 6 associated meningoencephalitis; and acute Epstein-Barr virus infection, early convalescent stage.

^2^Geographic zones based on Census Regions and Divisions of the United States, U.S. Department of Commerce Economics and Statistics Administration, U.S. Census Bureau

Real-time qPCR was performed using primers for a portion of the XMRV integrase gene [18] by a scientist who was blinded to the identity of the samples. The PCR assay could reliably detect 5 copies of XMRV DNA per reaction in the presence of 250 ng of cellular genomic DNA (or 20 copies of XMRV DNA/ug cellular DNA). Positive samples were defined as those having detectable DNA in the majority of replicates; at least 3 replicates were performed on each sample. Nine of 61 (15%) samples from patients with CFS were positive for XMRV DNA in the real-time qPCR assay. The mean DNA copy number of the positive samples was 21 copies per ug of cellular DNA, with a range of 9.9-37 copies per microgram DNA. There were no significant differences in the age, gender, or mean years of illness in XMRV-positive vs. XMRV-negative persons (Table 1). Patients with low levels of XMRV DNA were more likely to be from the Midwest and less likely to be from the Northeast. In contrast, none of the 18 subjects with idiopathic chronic fatigue, non-CFS viral disease, or healthy blood bank donors in the cohort was positive for XMRV. The difference between the PCR positivity for XMRV in patients with CFS versus patient controls or healthy blood donors in cohort 1 was not significant (p = 0.593). Therefore, the very weak signal (less than twice the lower limit of reliable detection) for XMRV in the minority of patients with CFS was not conclusively associated with this disease.

PCR was performed on patients with CFS that were positive for XMRV DNA using primers specific for mouse GAPDH or IAP sequences. All of the samples were negative for mouse GAPDH or IAP DNA; in contrast genomic DNA from mouse cells yielded the predicted size (140 bp for GAPDH and 300 bp for IAP DNA) band in the assay (data not shown).

We then measured the level of XMRV DNA in the blood from cohort 2 which included 50 healthy persons and 97 patients with chronic inflammatory diseases including 30 with rheumatoid arthritis, 20 with Behcet's disease, 10 with systemic lupus erythematosus, and 9 with cryopyrin-associated periodic syndromes (Table 2). All 97 samples were negative for XMRV DNA. To confirm that the genomic DNAs used did not contain any inhibitor that could account for lack of amplification, the RNAse P sequences were amplified using an RNAse P qPCR kit; all 226 samples were positive for RNAse P.

**Table 2:** Results of PCR for patients with chronic fatigue syndrome and controls.

Patient category	No. Subjects	qPCR Pos. for XMRV Integrase	qPCR Pos. for XMRV Env	Nested PCR Pos. for MLV-RV	Nested PCR Pos. for XMRV
Cohort 1					

CFS	61	9	0	61	0

Idiopathic chronic fatigue	6	0	0	6	0

Non-CFS virus disease*	3	0	0	3	0

Healthy donors	9	0	0	9	1

Cohort 2					

Rheumatoid arthritis	30	0	0	10**	ND

Behcet's disease	20	0	0	ND	ND

Systemic lupus erythematosus	10	0	0	ND	ND

Cryopyrin-associated periodic syndromes	9	0	0	ND	ND

Dermatomyositis	9	0	0	ND	ND

Polymyositis	7	0	0	ND	ND

Scleroderma	5	0	0	ND	ND

Inclusion body myositis	4	0	0	ND	ND

Other inflammatory diseases***	3	0	0	ND	ND

Healthy donors	50	0	0	ND	ND

*One patient each with chronic active hepatitis B virus infection; echovirus 6-associated meningoencephalitis, and acute Epstein-Barr virus infection, early convalescent stage

**Only 10 out of a total 30 samples from patients with rheumatoid arthritis were tested in the nested PCR assay

***Other inflammatory diseases: interstitial lung disease, monoclonal gammapathy of undetermined significance, undifferentiated connective tissue disease

We then tested DNA from all 226 samples in a real-time qPCR assay for a portion of the XMRV envelope gene [3]. This PCR assay could not detect fewer than 15 copies of XMRV per 250 ng of genomic DNA; therefore, it was less sensitive than the XMRV integrase PCR. All 226 DNAs were XMRV-negative using the XMRV envelope assay. These results are similar to prior results in which XMRV sequences could not be detected in samples from a CFS cohort from the United Kingdom [3].

### XMRV cannot be detected in PBMCs from patients with CFS by activation of the cells

These data prompted us to carry out further experiments on a subset of qPCR-positive and negative samples by looking for the amplification of XMRV proviral DNA after activation of patient PBMC by PHA and further stimulation with IL-2. No amplification was observed using the integrase based qPCR assay, including those that scored positive in the initial assay. In addition, repeated attempts to isolate and clone PCR-amplified DNA from a subset of the qPCR-positive samples were unsuccessful. This suggests that the samples that were weakly positive initially in the CFS patients may have been due to non-specific amplification of cellular DNA or a falsely positive signal.

### PBMCs from patients with CFS are not more likely to have a signal for MLV-related viruses than controls

Recently MLV-related virus DNA was detected in patients with CFS using nested PCR [2]. Initially we used Taq polymerase (Invitrogen) that does not contain the mouse monoclonal antibody present in Platinum Taq. However, no signal was observed in the patient or control samples. Therefore, we used Platinum Taq polymerase (the Taq polymerase used by Lo et al. [2], but the no template controls also amplified a DNA product. However, a new lot of Platinum Taq polymerase was selected which did not amplify a DNA product in the no template controls. When this Taq polymerase was used with genomic DNA template in a nested PCR assay, a 400 bp DNA was readily amplified from both CFS patients and non-CFS subjects (9 healthy blood donors, 10 patients with rheumatoid arthritis, and 9 other patients). The PCR amplification products from 14 CFS patients and 8 healthy donors were sequenced and aligned to sequences in Genbank using a BLAST search. We identified at least 5 distinctive *gag *sequences homologous to endogenous MLVs of the polytropic (Pmv), modified polytropic (Mpmv), or xenotropic (Xmv) subgroups (Table 3). The sequence from one control (healthy donor 2) is an MLV-like gag containing a region highly homologous to Mpmv MLV followed by sequence with lower homology to known MLVs. Most of these PCR products were also highly related to MLV-related sequences that were previously identified in samples from patients with CFS or prostate cancer [2,19,20]. In addition, a portion of human chromosome 1 was amplified from two CFS patients and one control sample. While the numbers of samples analyzed were small, MLV-related virus sequences were detected in both patients with CFS and in healthy controls indicating that the amplification of viral DNA was not specific for CFS. No PCR product was obtained in the absence of a template or using African green monkey (Vero) cells or telomerase-transformed rhesus fibroblasts (Telo-RF cells) grown in cell culture.

**Table 3:** Comparison of sequences amplified from healthy blood donor and CFS patient PBMC DNAs with sequences of endogenous MLVs and other patient derived sequences.

Source	Length (bp)	Related mouse sequence: Genbank number/ID	Type	Identity	Related human derived sequence Genbank number (identity)/ID
Healthy Donor#2	740	1-357 CU407131 Mouse chromosome 11	Mpmv	97% (346/356)	JF288884 (97%) XMRV HM630559 (97%) MLV-related virus
		
		411-534 AC161435 Mouse chromosome 8	Pmv	74% (80/108)	HM630559 (80%) MLV-related virus

Healthy Donor#4	472				AL356390 (99%) Human chromosome 1

CFS#1	338	CU407131 Mouse chromosome 11	Mpmv	96% (323/338)	JF288884 (96%) XMRV HM630559 (95%) MLV-related virus

CFS#2	437				AL356390 (99%) human chromosome 1

CFS#4	359	FJ544577 Mouse endogenous polytropic provirus clone 15 AC161435 Mouse chromosome 8	M/pmv Pmv	97% (327/336) 97% (327/336)	HM630560(97%) MLV-related virus

CFS#5	472				AL356390 (99%) Human chromosome 1

CFS#6	358	CU407131 Mouse chromosome 11	Mpmv	99% (284/288)	JF288884 (99%) XMRV HM990971(99%)Polytropic endogenous MLV

Abbreviations: CFS, chronic fatigue syndrome; Pmv, endogenous polytropic MLV; Mpmv, endogenous modified polytropic MLV; Xmv, endogenous xenotropic MLV; M/pmv, endogenous MLV of uncertain subtype

### Patients with CFS and a weak signal for XMRV do not have detectable antibody to XMRV

**Figure 1:**
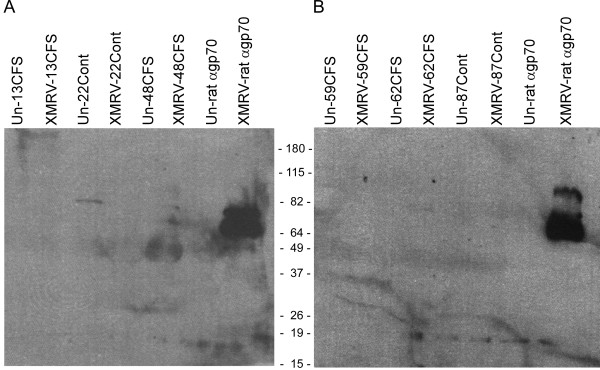
**Absence of antibody to XMRV gp70 in serum from patients with CFS**. XMRV gp70 protein was immunoprecipitated from XMRV-uninfected (Un) or XMRV-infected (XMRV) ferret cells using serum from subjects with (CFS) or without (Cont) CFS or with a rat monoclonal antibody to gp70. Immune complexes were separated on 4-20% SDS gels transferred to nitrocellulose membranes and immunoblotted with goat polyclonal antibody to XMRV gp70. (A) Sera from CFS patients 13 and 48, and control subject 22. (B) Sera from CFS patients 59 and 62 and control subject 87. The numbers between the blots are sizes of protein markers in kilodaltons.

Since Lombardi et al. [1] detected antibody to XMRV in many of their patients with CFS, we also looked for antibody in our patients. Immunoprecipitation of XMRV antigens from mock-infected or XMRV-infected ferret cells using serum from CFS patients and non-CFS controls, followed by immunoblotting with a polyclonal goat antibody to XMRV gp70 did not reveal the presence of antibodies to XMRV gp70 antigen (Figure 1). A positive control, in which an XMRV gp70-reactive rat monoclonal antibody 83A25 [21] was used to immunoprecipitate gp70, readily detected gp70 from XMRV-infected ferret cells (Figure 1A, B last lanes). A faint band co-migrating with gp70 was observed in the serum from one subject without CFS using either XMRV-infected 293T or ferret cells. The origin of this faint band was not further investigated. Similar results, showing lack of detectable antibody to XMRV, were also observed in virus-infected 293T cells (data not shown).

## Discussion

We initially detected very weak signals for XMRV in 15% of patients with CFS with a set of XMRV primers using real-time PCR, but failed to detect XMRV in patients with idiopathic chronic fatigue, chronic inflammatory disorders, other viral infections, or healthy controls. The difference in frequency of a weak PCR positive signal for XMRV for patients with CFS versus controls was not significant, thus, these results did not indicate a clear relationship between XMRV and CFS. Our repeated failure to isolate and clone XMRV sequences by PCR from the samples that were low positive in the real-time PCR assay, suggests that the low positive signals were false-positive artifacts, rather than contamination with mouse DNA. Tuke et al. [22] reported that Invitrogen Platinum Taq PCR Master Mix was contaminated with mouse DNA sequences; however, they did not detect murine sequences in Applied Biosystems Taq PCR Master Mix. Since we also used Applied Biosystems Taq PCR master mix for amplication of DNA by real-time PCR, this further supports the likelihood of a false positive signal as an artifact, rather than contamination with XMRV. We were also unable to detect XMRV in patients with CFS using a different set of XMRV primers or in PBMCs activated by PHA and further stimulated with IL-2 to amplify XMRV DNA. These latter results are in contrast to those of Lombardi et al. who found that activation of PBMCs from patients with CFS with PHA induced expression of virus proteins and infectious viral particles [1], which would be expected to amplify XMRV DNA.

Since the original report of Lombardi et al. [1] in which XMRV DNA was detected in 67% of CFS patients and 3.7% of controls, there are numerous reports from the United Kingdom [3,4], the Netherlands [5], China [6], Japan [12,23], and the United States [7-10,19,24] that did not detect XMRV DNA in patients with CFS. Reasons postulated for the difference in results include geography of the patients, contamination of samples or reagents with mouse DNA, sequence variation in XMRV, and definitions of CFS.

The blood samples we evaluated were collected from the NIH cohort of CFS patients during 1993-1995 and patients were predominantly from Midwestern and Southern United States, but also included patients from Western and Northeastern United States. Lombardi et al. studied patients from the 2006 to 2008, including some patients identified during an outbreak of CFS in 1984-1988, and their patients came from at least 11 states in various regions of the country [1,17]. Like our cohort, their patients were from diverse areas of the United States. Prior reports generally have studied more recent patients and most of the patients have been from more restricted locations in the United States or different geographic areas than those of the original report. Our study more closely resembles that of Satterfield et al. [24] who studied patients from multiple states of the continental United States, whereas other studies have reported patients from a single geographic area of the United States [8-11], two states [7], or other countries [3-6,23,25].

Using the nested PCR assay reported by Lo et al. [2], we detected MLV-related viral DNA in all human samples tested, regardless of whether they were from patients with CFS, inflammatory diseases, or normal controls. Sequence analysis showed that sequences in both CFS and healthy controls aligned with MLV sequences found in mice and reported in patients with CFS or prostate cancer [2,19,20]. In contrast, we did not detect MLV-related virus in monkey cell lines. While the nested PCR findings suggest that the human samples were contaminated with mouse DNA, specific testing for mouse genomic DNA (testing for both mouse GAPDH and multi-copy mouse IAP transposons) was negative. In addition, nested PCR of monkey cell DNA did not amplify MLV sequences. Thus, the MLV sequences might be due to contamination during DNA isolation (from the AllPrep DNA/RNA isolation kit), from Platinum Taq (as reported by Tuke et al. [22], or due to very low levels of MLV related sequences in human DNA. Using other procedures for isolating DNA and PCR, Satterfield et al. [24] did not detect MLV in CFS patients from 17 states in the United States.

Our serologic analysis by immunoprecipitation of gp70 from XMRV-infected 293T or ferret cells using CFS patient sera and sera from non-CFS control patients did not detect XMRV gp70 specific antibodies in patients with CFS. Our results are consistent with those of Erlwein et al. [25], Satterfield et al. [24], Knox et al. [9] and Shin et al. [10] who were unable to detect antibody to XMRV in patients with CFS.

Since this work was performed, Paprotka et al [15] and Knox et al [9] have shown that XMRV originated sometime between 1993 and 1996 from recombination between two endogenous MLVs during tumor passaging in mice, and that XMRV could not be detected in 43 patients who had previously been reported XMRV positive. Our findings, indicating no definitive evidence linking XMRV with CFS both by PCR and by antibody testing, support those of others that XMRV is not a cause of CFS. Thus, the search must continue for other etiologies for CFS.

## Conclusions

We did not find evidence linking XMRV to CFS by PCR of PBMCs or by immunoblotting of patient serum. Furthermore we did not find XMRV sequences in patients with connective tissue disorders including rheumatoid arthritis, Bechet's disease, and systemic lupus erythematosus.

## Methods

### Patients, controls, and DNA isolation

Subjects in cohort 1 with CFS, idiopathic chronic fatigue, non-CFS viral disease, and healthy blood donors were obtained from protocols approved by the Institutional Review Board (IRB) of the National Institute of Allergy and Infectious Diseases (NIAID). All patients signed written consents and blood had been obtained during 1993-1995. CFS was defined using Centers for Disease Control and Prevention criteria [16]. Idiopathic chronic fatigue was defined as clinically evaluated, unexplained chronic fatigue that failed to meet criteria for the chronic fatigue syndrome [16]. Healthy donors had signed consents on an NIAID approved protocol.

Since CFS has been associated with chronic immune activation, we tested a second group of patients, Cohort 2, which included patients with inflammatory diseases who had signed consents as part of protocols approved by the joint National Institute of Diabetes, Digestive and Kidney Diseases/National Institute of Arthritis and Musculoskeletal and Skin Diseases IRB.

Genomic DNA was extracted from 5-10 × 10^6 ^peripheral blood mononuclear cells (PBMCs) in patients from cohort 1 that had been cryopreserved in liquid nitrogen using an AllPrep DNA/RNA isolation kit (Qiagen, USA). DNA from PBMCs in patients from cohort 2 was extracted using various procedures.

### PHA-induction and IL-2 stimulation of PBMCs

PBMCs from a subset of cohort 1 were activated by 0.5 ug/ml of PHA-L (Roche Diagnostics), and after 72 hours, cells were cultured with 20 U/ml of recombinant IL-2 (Biological Resource Branch, NCI) and subcultured every 3-5 days as described earlier [1]. Genomic DNA was prepared on day 8 from IL-2-stimulated cells using an AllPrep DNA/RNA isolation kit as described above.

### Real-time quantitative PCR of XMRV DNA

A 121 bp region from the XMRV integrase gene was amplified by real-time quantitative PCR (qPCR) using primers 4552F and 4673R and a 5'FAM and 3'TAMRA labeled probe 4572 as described previously [18]. A second real-time qPCR assay for a 71 bp fragment from the XMRV envelope gene used primers 6173 env F, 6173 env R, and 5'-FAM and 3'-TAMRA labeled 6173 envelope probe as described previously [3].

Reaction mixtures for qPCR contained 1X TaqMan Universal PCR Master Mix (Applied Biosystems), 1 uM each of primer, 250 nM of probe and 250 ng of genomic DNA in a total volume of 25 ul. All PCR reactions were performed with duplicate samples. Reactions were performed using an ABI 7500 real-Time PCR System (Applied Biosystems) with the following conditions: 50°C for 2 min, 95°C for 10 min, 40 cycles at 95°C for 20 sec and 60°C for 1 min. A standard curve consisting of 10-fold serial dilutions (5-50,000 copies) of XMRV proviral DNA VP62 [26; a gift from F. Ruscetti, NCI] spiked with 250 ng of denatured salmon sperm DNA was amplified using identical conditions to quantify the presence of XMRV DNA in the human samples. The assay can reliably detect 20 copies of XMRV DNA/ug of cellular DNA. To ensure that no inhibitor was present, the single copy RNAse P gene was amplified from each 250 ng DNA sample using a TaqMan RNAse P Detection Reagent kit (Applied Biosystems).

### PCR amplification for XMRV and MLV-related virus DNA

Nested PCR of MLV-related virus and XMRV gag sequences was performed as described by Lo et al. [2]. XMRV DNA was amplified for 40 cycles using primers 419F and 1154R with 50 ng of genomic DNA and 1 U of Platinum Taq polymerase (Invitrogen) followed by 45 cycles in the 2^nd ^round using either primers GAG-1F and GAG-1R for XMRV DNA, or primers N116 and N117 for MLV-related virus [2]. Multiple controls without template were run in each PCR experiment.

### PCR amplification of mouse DNA

The presence of mouse DNA was tested by PCR for mouse GAPDH which is present at 2 copies/mouse genome as described earlier [26]. Low level contamination of mouse DNA was tested by amplification of intracisternal A particle (IAP) transposons which are present at ~1000 copies/mouse genome as described previously [11]. Serial dilutions of mouse DNA were used as positive controls, and PCR products were analyzed by electrophoresis on agarose gels.

### Immunoprecipitation and immunoblotting for XMRV gp70 protein

The presence of antibodies to XMRV in human serum samples was tested by immunoprecipitating XMRV antigens from XMRV-infected or mock infected 293T or ferret cell extracts. Ferret cells were found to yield a higher level of infection than human cell lines (e.g. HeLa, 293T cells). 293T cells were obtained from the American Type Culture Collection (Manassas, VA) and MA-139 ferret cells [27] were a gift from Janet Hartley, NIAID, Bethesda, MD. Briefly, about 40 ul of cell extracts were mixed with 280 ul of 1× RIPA buffer (10 mM Tris-HCl, pH 8.0, 150 mM NaCl, 1 mM EDTA, 1.0% NP-40, 0.5% deoxycholate and 0.1%SDS). Human serum (20 ul) or cell culture filtrate containing rat monoclonal antibody 83A25 [19] to XMRV gp70 (20 ul) was added. The mixture was incubated on ice for 1 h. Then 40 ul of a 50% suspension of protein-G-Sepharose (Sigma-Aldrich) was added and the antigen-antibody reaction was allowed to proceed for 12-16 h at 4°C in a rotator. Immune complexes were purified by washing the beads 3-4 times with 1× RIPA buffer and boiled for 3 min in 1× SDS-PAGE loading buffer (Quality Biologicals). Proteins were separated on 4-20% Tris-glycine SDS-PAGE gels (Invitrogen), transferred to nitrocellulose membranes, and incubated with a goat polyclonal antibody to XMRV gp70 (produced by Viromed Biosafety Laboratories, Camden NJ) at a dilution of 1:3000. After washing, the membrane was incubated with HRP-conjugated donkey anti-goat antibody (Santa Cruz Biotechnology).

### Statistics

Fisher's exact test was used to test the significance between groups.

## List of abbreviations

XMRV: xenotropic murine leukemia virus-related virus; CFS: chronic fatigue syndrome: MLV: murine leukemia virus; IRB: institutional review board; NIAID: National Institute of Allergy and Infectious Diseases; PBMCs: peripheral blood mononuclear cells; IAP; intracisternal A particle; Pmv, polytropic MLV; Mpmv, modified polytropic MLV; Xmv, xenotropic MLV.

## Competing interests

The authors declare that they have no competing interests.

## Authors' contributions

All authors read and approved the final manuscript. MAA performed the experiments and wrote the paper. JKD and SES conducted clinical research and contributed patient samples and provided demographic data. CAK provided reagents for XMRV and advice on XMRV. RGM and FWM provided control samples. JIC designed the experiments and wrote the paper.
